# High Endurance Elite Athletes Show Age-dependent Lower Levels of Circulating Complements Compared to Low/Moderate Endurance Elite Athletes

**DOI:** 10.3389/fmolb.2021.715035

**Published:** 2021-09-23

**Authors:** Shamma Al-Muraikhy, Manjunath Ramanjaneya, Alexander S. Dömling, Ilham Bettahi, Francesco Donati, Francesco Botre, Abdul-Badi Abou-Samra, Maha Sellami, Mohamed A Elrayess

**Affiliations:** ^1^ Biomedical Research Center, Qatar University, Doha, Qatar; ^2^ Department of Drug Design, University of Groningen, Groningen, Netherlands; ^3^ Qatar Metabolic Institute, Hamad Medical Corporation, Doha, Qatar; ^4^ Translational Research Institute, Hamad Medical Corporation, Doha, Qatar; ^5^ Laboratorio Antidoping, Federazione Medico Sportiva Italiana, Rome, Italy; ^6^ Physical Education Department, College of Education, Qatar University, Doha, Qatar

**Keywords:** complements, endurance, elite athletes, aging, inflammatory cytokines, telomere length

## Abstract

**Introduction:** Aerobic exercise activates the complement system in the peripheral blood. However, the effect of age and high intensity endurance training on the levels of circulating complements and sassociated inflammatory cytokines, oxidative stress markers and cellular aging remains unknown.

**Methods:** In this study, serum samples from 79 elite athletes who belong to high (*n* = 48) and low/moderate (*n* = 31) endurance sports and two age groups (below 30 years old, n = 53, and above 30 years old, *n* = 26) were profiled for 14 complements. Linear models were used to assess differences in complements levels between sport and age groups. Spearmann’s correlation was used to assess the relationship among detected complements and proinflammatory cytokines, oxidative stress markers and telomere lengths.

**Results:** High endurance elite athletes exhibited significantly lower levels of circulating C2, C3b/iC3b and adipsin complements than their age-matched low/moderate endurance counterparts. Levels of C2, adipsin and C3b/iC3b were positively correlated with most detected complements, the pro-inflammatory cytokines TNF-alpha and IL-22 and the anti-oxidant enzyme catalase. However, they were negatively correlated with telomere length only in younger elite athletes regardless of their sport groups. Furthermore, high endurance elite athletes showed significantly lower concentrations of C3b/iC3b, C4b, C5, C5a, C1q, C3, C4, factor H and properdin in younger athletes compared to their older counterparts.

**Conclusion:** Our novel data suggest that high endurance elite athletes exhibit age-independent lower levels of circulating C2, C3b/iC3b and adipsin, associated with lower inflammatory, oxidative stress and cellular aging, as well as lower levels of 10 other complements in younger athletes compared to older counterparts. Assessing the effect of various levels of endurance sports on complements-based immune response provides a better understanding of exercise physiology and pathophysiology of elite athletes.

## Introduction

As part of the innate immune response, the complement system protects against pathogens in the absence of the adaptive immunity (Medzhitov and Janeway, 1998) and links the innate and acquired immune responses (Carroll, 2000). It offers systemic surveillance and protection by chemotaxis-mediated phagocytosis, scavenging pathogens as well as necrotic and apoptotic debris. Complements can also mediate cell activation, regenerative processes, and humoral and cell-mediated immune responses (Schifferli et al., 1986; Ricklin et al., 2010). The complement system includes 50 proteins that constitute around 15% of the globulin fraction (Carroll, 2000; Walport, 2001). Three main pathways can activate the complement system: classical, lectin, and alternative. The classical pathway is triggered by antigen–antibody immune complexes, apoptotic and necrotic cells and by acute phase proteins such as C-reactive protein. The lectin pathway uses mannose-binding lectins and ficolins to identify patterns of carbohydrate ligands found on the surface of microorganisms. The alternative pathway is constitutively active at low levels in the normal host in preparation for rapid and robust activation upon stimulation (Noris and Remuzzi, 2013).

Each component of the complement system offers a unique function in response to different stimuli. In the classical pathway, C2 is cleaved into C2a and C2b, followed by the fusion between C2a and C4b components, forming the classical-C3 convertase with proteolytic activity (Sarma and Ward, 2011; Prohászka et al., 2018). C3 complement participates in both classic and alternative pathways and its deficiency can cause impairment of the immune response and enhanced risk of infection (Matsuyama et al., 2001). C3 protein cleavage into C3b component is mediated by C3 convertase, which is also shared by the lectin activation pathway. The overstimulation of the activity of the complement system could harm the body, causing serious diseases such as organ rejection, asthma, multiple sclerosis, sepsis, or Alzheimer’s disease (Sarma and Ward, 2011). Previous studies have shown that complement C3 is involved in the aging process (Wu et al., 2020) and that secretion of complement C1q is increased with aging in association with elevated vascular smooth muscle cells proliferation (Hasegawa et al., 2019). The alternative pathway is more dominant compared to the classical one (Sarma and Ward, 2011). Adipsin (complement factor D) is secreted by macrophages, monocytes and adipocytes and controls the alternative complement pathway and the generation of C3a (Gómez-Banoy et al., 2019; Ohtsuki et al., 2019). In obesity, adipsin levels correlate with metabolic disease (Wang et al., 2019) and constitutes a good predictor of death in patients with coronary artery disease (Ohtsuki et al., 2019; Liu et al., 2021) and risk of nonalcoholic fatty liver (Qiu et al., 2019).

The role of the complement system in modulating disease progression in systemic autoimmunity and primary immune-deficiencies is well established (Ricklin et al., 2010; Skattum et al., 2011; De Cordoba et al., 2012; Holers, 2014). Its involvement in post-effort immunity is also documented (Mashiko et al., 2004; Karacabey et al., 2005). However, its role in the chronic exercise immune response is yet to be determined. A recent study has suggested that aerobic exercise can trigger various immune responses in active young men via activating the alternative pathway (Kostrzewa-Nowak et al., 2020). Whereas moderate intensity regular exercise stimulates the immune system, high intensity exercise without sufficient recovery could reduce the immune response and enhance risk of infections (Calabrese and Nieman, 1996; Nieman, 1997; Pedersen and Hoffman-Goetz, 2000; Gleeson et al., 2012; Peake et al., 2017a; Peake et al., 2017b). Hence, intensive physical activity can stimulate or suppress the immune response based on many factors such as age and fitness level, triggering oxidative stress and release of cortisol, catecholamines, insulin like growth factor and heat shock proteins (Nieman, 1997; Liu et al., 2018). The main aim of this study was to examine the impact of two types of endurance sports (low/moderate vs high) in elite athletes on the complement system and post-exercise immune response in younger (less than 30 years old) and older (more than 30 years old) healthy athletes. Profiling complements in elite athletes who belong to different exercise intensity and age groups could help in the understanding the impact of chronic exercise and aging on the immune response.

## Methods

### Cohort

Blood samples from 79 consented elite athletes (68 males and 11 females), defined as those participating in national or international sports events and tested for doping abuse by accredited anti-doping laboratories, who belong to different sport disciplines (Table 1) were previously collected for doping analysis of growth factors at anti-doping laboratory in Italy (FMSI). Spare sera collected for anti-doping human growth hormone testing were utilized for complements profiling. Briefly, samples were collected by doping officers in serum separator tubes, then delivered to the anti-doping laboratory within 36 h under cooling conditions. Once received, samples were immediately centrifuged to separate the serum and then stored at −20°C until analysis. Following published criteria (Mitchell et al., 2005; Al-Khelaifi et al., 2018a; Al-Khelaifi et al., 2018b; Al-Khelaifi et al., 2019), participants were divided into two intensity groups based on their sports type: low/moderate endurance sports (*n* = 48, 43 males and five females) and high endurance sports (n = 31, 25 males and six females). Participants were also divided into two age groups: less than 30 years old (n = 53) and above 30 years old (*n* = 26). This study is performed in line with the World Medical Association Declaration of Helsinki–Ethical Principles for Medical Research Involving Human Subjects. All protocols were approved by the Institutional Research Board of Qatar University (QU-IRB 1277-E/20). All participants consented for the use of their samples for research.

**TABLE 1:** Classification of participants (males: M and females: F) according to the endurance intensity of their respective sports.

Low/Moderate endurance (*n* = 48)	High endurance (*n* = 31)
1 Cricket (M), 1 Equestrian (M), 1 Golf (M), 1 Powerboating (M), 1 Sport climbing (M), 30 Football (30M), 1 Athletics-throws (M), 2 Bobsleigh (1M, 1F), 2 Gymnastics (2F), 1 Luge (M), 2 Sport climbing (1M, 1F), 1 Volleyball (F), 4 Wrestling (4M)	2 Cycling-track endurance (2M), 4 Cycling-cross (2M, 2F), 5 Triathlon (4M, 1F), 12 Athletics-long distance (11M, 1F), 4 Cycling-track endurance (2M, 2F), 2 Cycling-road (2M), 2 Triathlon (2M)

### Human Complement Related Protein Measurements

MILLIPLEX MAP Kit Human Complement Magnetic Bead Panels one and 2 (HCMP1MAG-19K and HCMP2MAG-19K) were used to measure levels of complements in the sera of elite athletes according to manufacturer’s instructions (Merck Millipore, United States). Sensitivity and accuracy levels can be found under the physicochemical information in the manufacturer’s website. Serum samples were diluted 200 times for complement panel one containing C2, C4b, C5, C5a, C9, Factor D, and Mannose-Binding Lectin and 40,000 times for complement panel two containing C1q, C3, C3b/iC3b, C4, Factor B, Factor H and Properdin. Five parameters logistic regression algorithms built into the Bioplex manager six software were used to assess complement levels in reference to standards. Analysis was conducted using a Bioplex-200 instrument according to manufacturer’s instructions (BIO-RAD, Hertfordshire, United Kingdom).

### Cytokine profiling

Human CorPlex Cytokine one Array 10-Plex (116-7BF-1-AB) was used to simultaneously profile 10 cytokines, including IL-12p70, IL-1β, IL-4, IL-5, IFN-γ, IL-6, IL-8, IL-22, TNF-α, IL-10, according to manufacturer’s instructions (Quanterix, United States) and recently described (Sellami et al., 2021a).

### Measurement of anti-oxidative stress enzymatic activities

Colorimetric activity assays (EIACATC and EIASODC) were used to measure the activities of superoxide dismutase and catalase according to manufacturer’s instructions (ThermoFisher Scientific, United States) and recently described (Sellami et al., 2021a).

### Measurement of telomere length

DNA was extracted from whole blood using DNeasy Blood and Tissue kit according to manufacturer’s instructions (Qiagen, Germany). Nanodrop was used to assess the concentration/quality of DNA. The average telomere lengths in extracted DNA samples were assessed using Absolute Human Telomere Length Quantification qPCR Assay Kit according to manufacturer’s instructions (ScienCell, United States). The kit includes telomere primer set that amplifies telomere sequences, a single copy reference region for data normalization and a reference genomic DNA sample with known telomere length as a reference for calculating the telomere length of target samples.

### Statistical analysis

Comparisons were performed using *t*-test, Wilcoxon–Mann–Whitney, 1-way ANOVA, or linear models as appropriate using IBM SPSS statistics 21. Linear regression models were used when analyzing differences in complements levels among different age and intensity groups by considering gender as a potential confounder as females were over represented in the high endurance group. Data were presented as mean ± standard deviation (SD) for parametric data, median (interquartile range, IQR) for non-parametric data verified by skewness and kurtosis test.

## Results

### Differences in the levels of complements between athletes who belong to low/moderate versus high endurance sports

**FIGURE 1:**
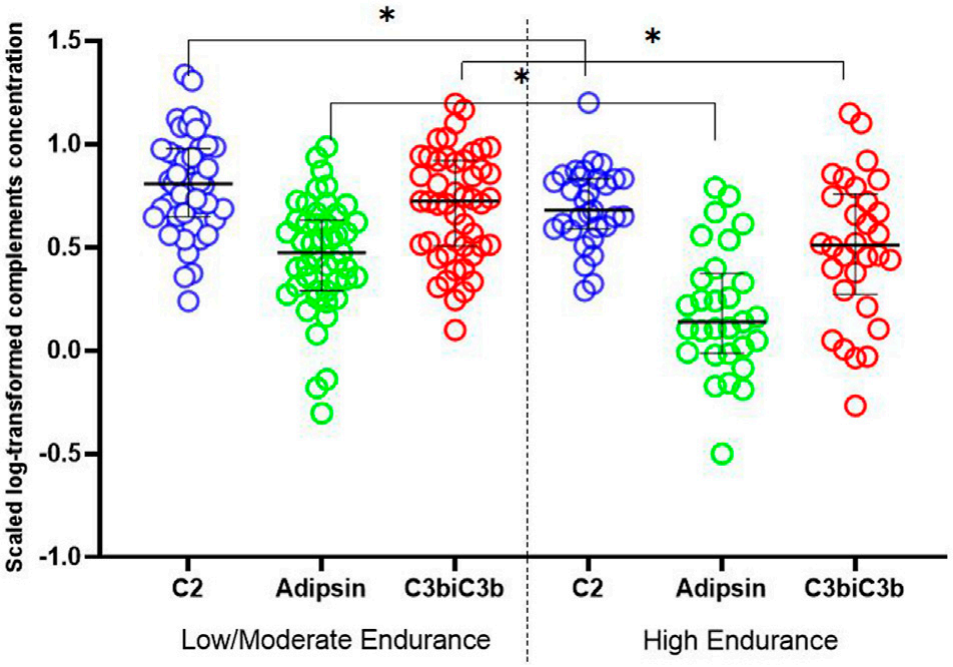
Dot plot exhibiting differences in complements levels between high and mild/moderate endurance sports. Independent sample *t*-test was used to compare scaled log-transformed cytokine levels (*y* axis) in different sport intensity groups. Data are presented as median and IQR. * <0.05.

Among the detected complements, C2, adipsin and C3b/iC3b showed significantly lower levels in athletes who belong to high endurance sports compared to age-matched athletes who belong to low/moderate endurance sports (Table 2; Figure 1). Similarly, there were lower levels of factor B, factor H and properdin in high endurance athletes compared to low/moderate endurance athletes, but differences did not reach statistical significance (*p* = 0.1).

**TABLE 2:** Comparing levels of complements between high versus low/moderate elite endurance athletes.

—	All athletes N = 79	Low/Moderate endurance athletes *n* = 38	High endurance athletes *n* = 41	*p* value (nominal)
Age (years)	26.4 (7.5)	26.4 (7)	26.4 (8.5)	0.99
Complement C2 (µg/ml)	5.8 (4.0, 8.0)	6.4 (4.4, 9.5)	4.8 (3.9, 6.8)	0.02
Adipsin (µg/ml)	2.3 (1.4, 4.1)	3.0 (1.9, 4.3)	1.4 (1.0, 2.4)	<0.001
Complement C3b/iC3b (mg/ml)	4.6 (2.8, 7.2)	5.3 (3.2, 8.3)	3.2 (1.9, 5.7)	0.03
Complement C4b (µg/ml)	9.3 (5.1)	9.6 (4.7)	8.8 (5.8)	0.52
Complement C5 (µg/ml)	18.3 (11.6)	19.4 (9.8)	16.6 (13.9)	0.29
Complement C5a (µg/ml)	1.7 (1.1, 2.5)	1.9 (1.1, 2.5)	1.5 (1.1, 2.6)	0.85
Mannose-binding lectin (µg/ml)	1.2 (0.5, 2.7)	1.2 (0.5, 3.0)	0.8 (0.4, 1.7)	0.31
Complement factor-1 (µg/ml)	17.6 (10.1, 30.9)	20.2 (12.3, 32.7)	13.8 (7.4, 24.6)	0.24
Complement- C1q (µg/ml)	37.6 (21.5)	39.1 (19.7)	35.1 (24.4)	0.43
Complement C3 (µg/ml)	275.4 (180.1)	270.1 (170.6)	283.6 (196.5)	0.75
Complement C4 (µg/ml)	129.4 (78.0)	136.5 (76.2)	117.5 (81.0)	0.31
Factor-B (µg/ml)	79.6 (43.4)	86.1 (40.6)	69.4 (46.5)	0.1
Factor-H (µg/ml)	120.3 (63.2)	129.7 (58.5)	105.2 (6.8)	0.1
Properdin (µg/ml)	14.7 (8.5)	16.0 (7.9)	12.8 (9.0)	0.1

Data are presented as mean (SD) for normally distributed data or median (IQR) for skewed data. Samples were measured in duplicates. Comparisons were performed using *t*-test or Wilcoxon–Mann–Whitney as appropriate.

### Correlations between C2, adipsin and C3b/iC3b complements and other complements, inflammatory cytokines and oxidative stress markers

**FIGURE 2:**
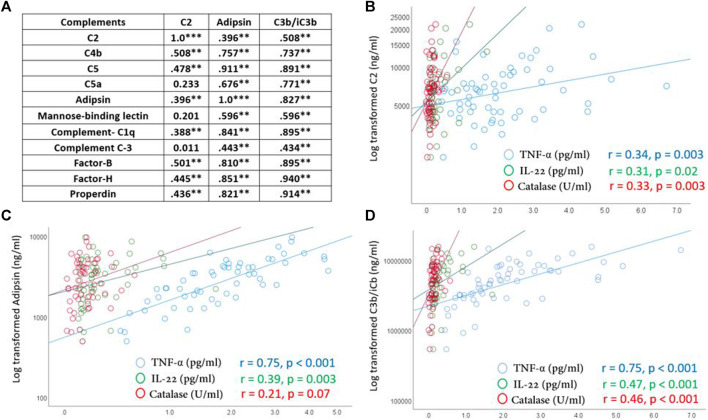
Correlations between C2, adipsin and C3b/iC3b complements and other detected complements **(A)** and inflammatory cytokines and antioxidant enzyme catalase **(B–D)**. Correlations were made using spearman’s correlation analysis. Correlation coefficient (r) and significance (*p ≥ 0.05, **p ≥ 0.01, ***p ≥ 0.001) are indicated.

Levels of inflammatory cytokines and oxidative stress markers were recently published (Sellami et al., 2021b). There were significant positive correlations between C3, adipsin and C3b/iC3b and other measured complements in all participants (*n* = 79) regardless of sport intensity or age groups (Figure 2A). There were also positive correlations between C2, adipsin and C3b/iC3b complements and the pro-inflammatory cytokines TNF-α and IL-22 and the anti-oxidant enzyme catalase (Figures 2B–D).

### Differences in complement levels among athletes who belong to different age groups in low/moderate and high endurance elite athletes

**FIGURE 3:**
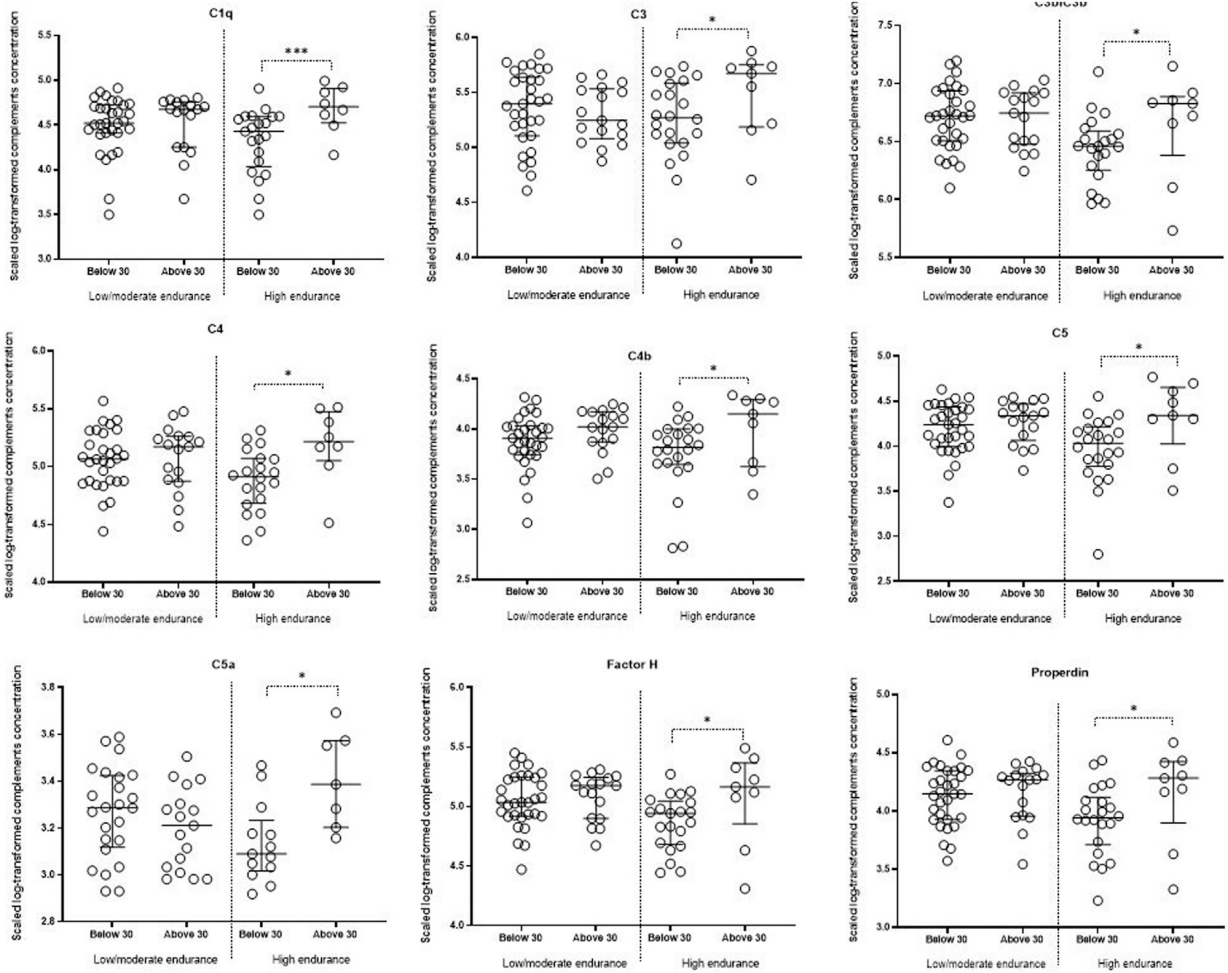
Comparing complements between different age groups in low/moderate and high endurance elite athletes. Linear models incorporating the interaction term (age versus sport intensity), followed by contrast analysis, were used to compare scaled log-transformed cytokine levels (*y* axes) in different sport intensity groups. Data are presented as median and IQR. * <0.05.

Linear models revealed that C3b/iC3b, C4b, C5, C5a, C1q, C3, C4, factor H and properdin were significantly lower in younger high endurance athletes compared to their older counterparts (Table 3; Figure 3). There were no significant differences in the levels of complements in low/moderate elite endurance athletes between the two age groups (Table 3).

**TABLE 3:** Comparing complements between different age groups in low/moderate and high endurance elite athletes.

	Low/Moderate endurance			High endurance		
	Below 30	Above 30	*p* value (nominal)	Below 30	Above 30	*p* value (nominal)
Age (years)	22 (2.6)	34.4 (4.9)	<0.01	21.8 (3.6)	37.6 (6.3)	<0.01
Complement C2 (µg/ml)	6.5 (4, 9.8)	6.4 (4.5, 9)	0.97	5.1 (3.9, 6.7)	4.6 (3.7, 7.2)	0.84
Adipsin (µg/ml)	2.5 (1.9, 3.8)	4.1 (2.4, 5.2)	0.16	1.3 (1, 1.9)	3.4 (1, 4.7)	0.17
Complement C3b/iC3b (mg/ml)	5.3 (3.2, 8.6)	5.6 (3, 8.3)	0.8	2.9 (1.8, 3.9)	6.7 (2.9, 7.7)	0.03
Complement C4b (µg/ml)	8.9 (4.7)	10.8 (4.4)	0.16	7.1 (4)	12.9 (7.7)	0.06
Complement C5 (µg/ml)	18.5 (10)	21.1 (9.4)	0.4	12 (8.1)	27.8 (18.9)	0.04
Complement C5a (µg/ml)	1.9 [1.3, 2.7]	1.6 (1.1, 2.2)	0.22	1.2 (1, 1.7)	2.4 (1.6, 3.7)	0.04
Mannose-binding lectin (µg/ml)	1.2 (0.6, 3)	1.7 (0.4, 2.9)	0.72	0.7 (0.4, 1.3)	2.4 (0.4, 5)	0.09
Complement factor-1 (µg/ml)	17.8 (12.1, 31.8)	23.5 (12.8, 36.6)	0.64	11.1 (7.4, 16.9)	26.2 (9.5, 44.8)	0.13
Complement- C1q (µg/ml)	38.1 (20)	40.9 (19.5)	0.64	27.3 (18.2)	55.4 (28)	<0.01
Complement C-3 (µg/ml)	291 (188.6)	232 (12.8)	0.26	235.7 (159.8)	400.6 (236.7)	0.03
Complement C4 (µg/ml)	133.9 (76.6)	141.1 (77.6)	0.76	89.4 (50.3)	187.6 (102.9)	0.03
Factor-B (µg/ml)	86.4 (42.3)	85.6 (38.6)	0.94	55.7 (30.9)	102.9 (61.9)	0.06
Factor-H (µg/ml)	128.2 (63.5)	132.4 (49.6)	0.82	83.4 (40.6)	156 (93.2)	0.05
Properdin (µg/ml)	15.7 (8.5)	16.4 (7.0)	0.78	10.4 (6.7)	18.7 (11.4)	0.02

Data are presented as mean (SD) for normally distributed data or median (IQR) for skewed data. Samples were measured in duplicates. Comparisons were performed using linear models incorporating the interaction term (age versus sport intensity), followed by contrast analysis.

### Correlations between complements and telomere length in two age groups

**FIGURE 4:**
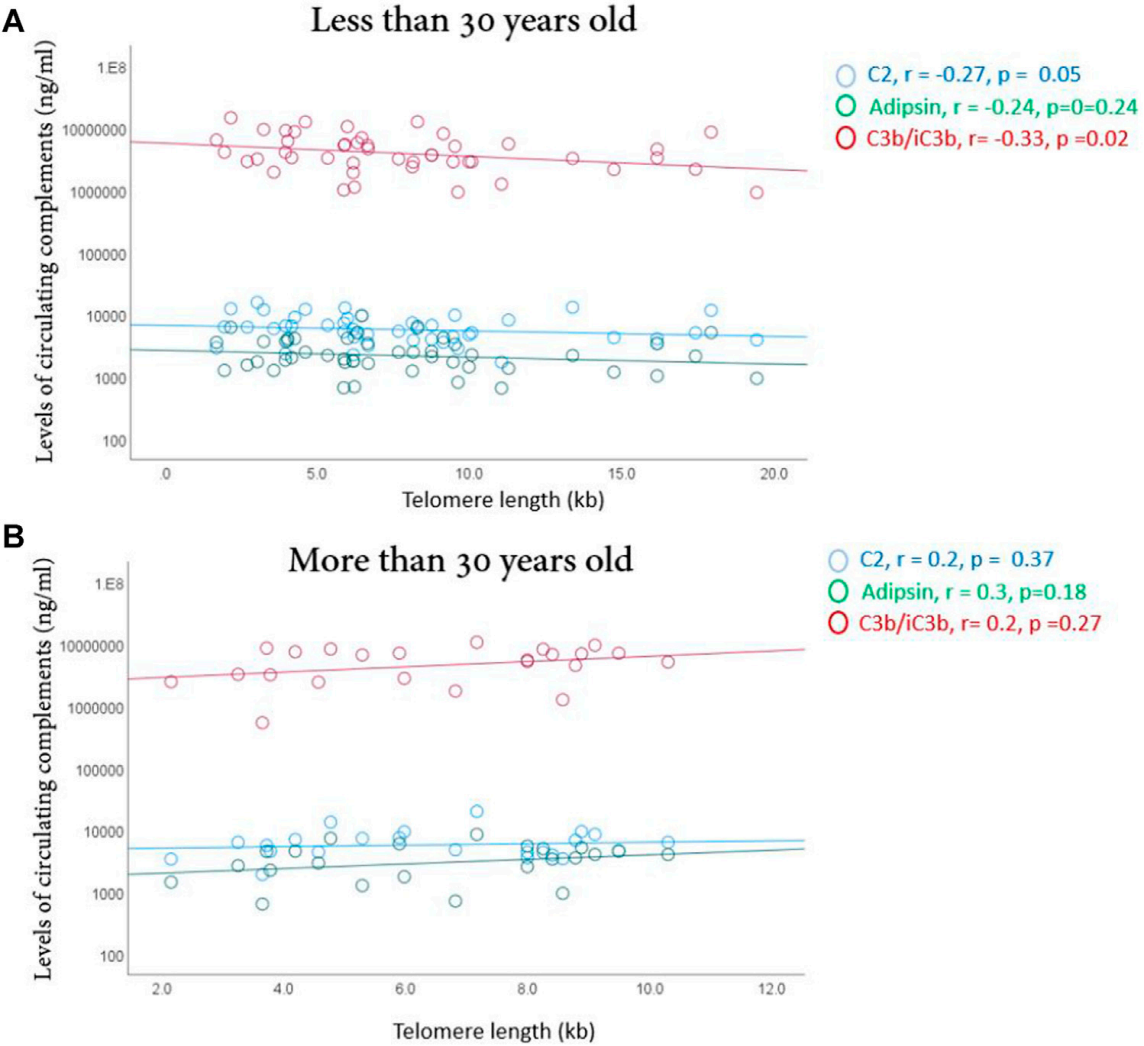
Correlation between telomere length and the complements (C2, adipsin and C3b/iC3B) in younger **(A)** and older **(B)** elite athletes. Correlations were made using spearman’s correlation analysis. Correlation coefficient (r) and significance are indicated.

There were significant negative correlations between telomere lengths and C2 (R = −0.27, *p* = 0.05) and C3b/iC3b (R = −0.34, *p* = 0.02) only in younger athletes (Figure 4), suggesting less cellular aging of blood cells in younger, but not older, high endurance elite athletes.

## Discussion

The effect of physical activity on the immune system varies according to the type, duration and intensity of the exercise (Nieman and Wentz, 2019). These effects are mediated by complex mechanisms that include hormonal, metabolic and psycho-neural changes (Silverman and Deuster, 2014). Short-term low/moderate physical activity with intensity <60% VO_2_max can modulate the immune response and enhance athlete immunity (Cannon, 1993; Sohail et al., 2020). Conversely, high endurance exercise with intensity greater than >70% VO_2_max could contribute to lowering the athletes’ immunity (Cannon, 1993; Mackinnon, 1994). As part of the immune response, acute physical activity can also activate the alternative complement pathway in an intensity and duration-dependent manners (Kostrzewa-Nowak et al., 2020), however the effect of long-term exercise, such as the one practiced by elite athletes on circulating complement levels remains to be investigated. In this study, circulating levels of 14 complements were profiled in the serum samples of 79 elite athletes who belong to high and low/moderate endurance sports and two age groups (below and above 30 years old). This age threshold was previously described as a discontinuation stage entailing elite athletes' transition out of competitive sports (Wylleman and Reints, 2010). Our emerging novel data suggest that high endurance elite athletes exhibit age-dependent and independent lower levels of specific complements associated with lower inflammatory, oxidative stress and cellular aging markers.

### Low levels of circulating C2, C3b/iC3b and adipsin complements in high endurance athletes

**FIGURE 5:**
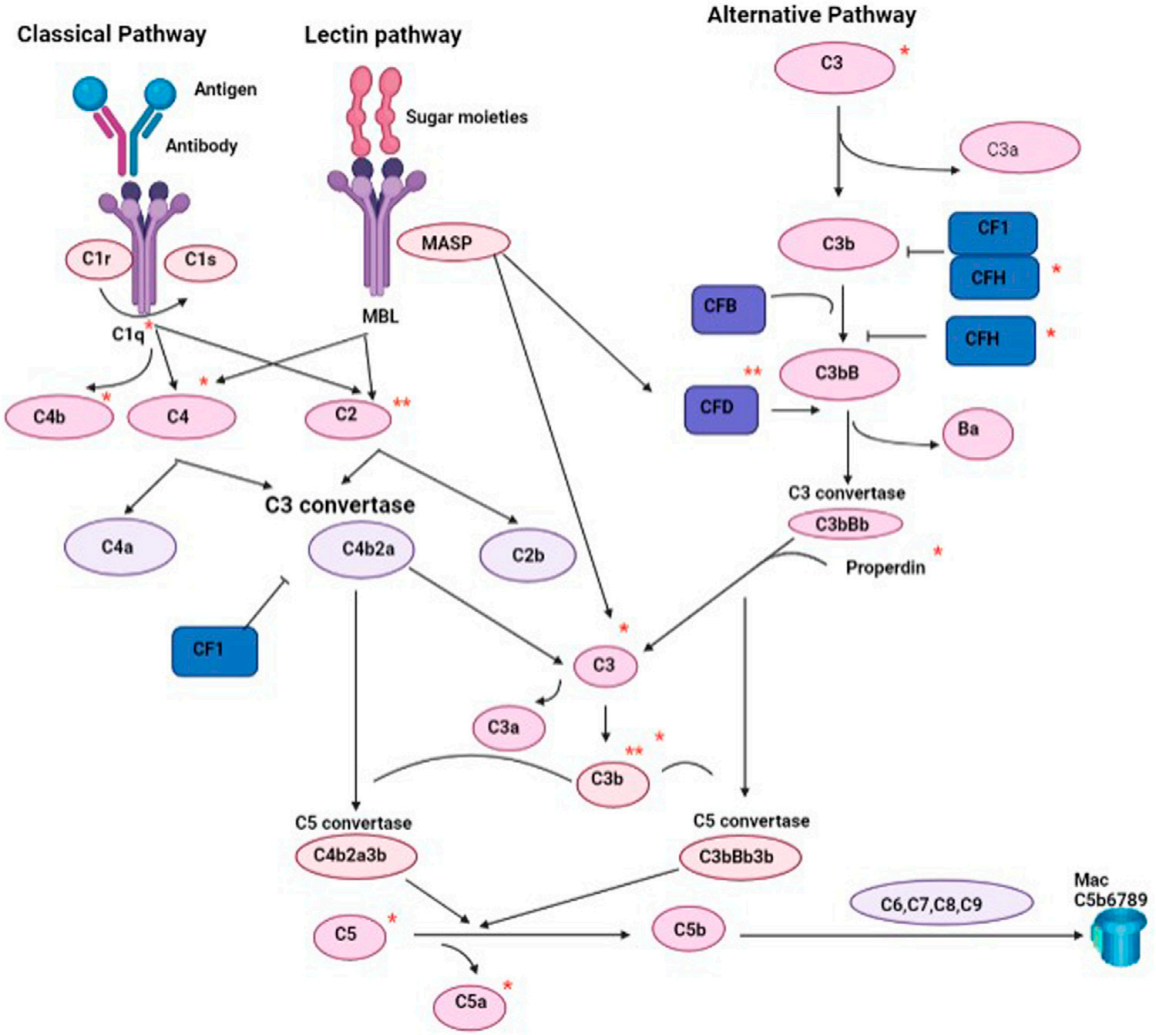
A schematic representation of complement pathways highlighting complements that were reduced in high endurance athletes (**) and those reduced in younger high endurance athletes (*). CF (complement factor). CFD (adipsin).

The main finding of this study was that athletes who belong to high or low/moderate endurance sports exhibit a differential profile of complement system. Athletes who belong to high endurance sports showed reduced activation of C2, C3b/iC3b and adipsin compared to the age-matched low/moderate endurance athletes. The complement C2 is a serine protease that provides catalytic activity to the C3 convertase of classical pathway of complements (Figure 5). The lower secretion of C2 in high endurance falls in line with the previously reported lower levels in long distance running athletes compared to volleyball players who belong to moderate endurance sports (Saygin et al., 2006). The complement C3 as well as the breakdown products C3b and iC3b, play a central role in the activation of the complement system, which is required for both classical and alternative complement activation pathways (Figure 5) (Noris and Remuzzi, 2013). Previous studies have demonstrated that acute endurance effort (20 m shuttle run test) triggers a decrease in the post-test C3, but increase in iC3b and C2 in 15–17 year old active males. On the other hand, repeated speed ability test caused a decrease in the post-test C2 for younger participants only, but an increase in the recovery C3a levels (Kostrzewa-Nowak et al., 2020). Differences in C3 responses to endurance between reported data and our study may reflect the difference in the duration (acute or chronic) or intensity (recreational or professional) of exercise between participants of the two studies. However, both data suggest that exercise can indeed trigger alterations in C2, C3b and iC3b levels in an intensity-dependent manner. The impact of lower levels of these complements on the immune response, general health and performance of elite high endurance athletes remains to be determined.

Our data also indicated lower adipsin levels in high endurance athletes. Adipsin, also known as complement factor D, is one of the major proteins secreted by adipocytes for maintaining adipose tissue homeostasis and increasing insulin secretion in response to glucose (Tafere et al., 2020). It also plays a critical role in mediating the rate-limiting step of the alternative pathway of complement activation through the formation of the C5-C9 membrane attack complex and the generation of a number of signaling molecules including the anaphylatoxins C3a and C5a (Figure 5) (Xu et al., 2001; Ricklin et al., 2010). It has been implicated in increased risk of models of ischemia reperfusion (Stahl et al., 2003), and sepsis (Dahlke et al., 2011). Lower plasma adipsin concentrations are described in animals and patients with type 2 diabetes mellitus, since its presence is important for improving hyperglycemia by preserving β-cell survival (Tafere et al., 2020). Therefore, the implication of lower levels of adipsin in high endurance elite athletes could indicate higher risk of metabolic disease as previously suggested (Al-Khelaifi et al., 2019), although further studies are required to investigate the functional relevance of lower adipsin.

### Positive correlations between C2, adipsin, C3b/iC3b and other complements, TNF-α, IL-22 and catalase

Significant positive correlations were identified between levels of the three complements that were reduced in high endurance athletes (C2, adipsin and C3b/iC3b) and other detected complements of the classical, lectin, and alternative pathways. These correlations are expected since they could simply reflect the identical nature of these complements caused by their duplicated genes that have diverged in sequence (Janeway et al., 2001). Furthermore, positive correlations were also seen between C2/adipsin/C3b/iC3b and the pro-inflammatory cytokines TNF-α and IL-22. Previous reports have indicated that T helper cell subset 22 (Th22) is characterized by a combinatorial secretion of IL-22 and TNF-α, and that IL-22 increases the TNF-α-dependent induction and secretion of several immune-modulatory molecules including complements (Eyerich et al., 2011). Such synergistic action could explain the positive correlation between these cytokines and C2, adipsin, C3b/iC3b. The production of catalase is increased at higher levels of exercise in order to compensate for the insufficient clearance of hydrogen peroxide by glutathione peroxidase (Finsterer, 2012). The positive correlation between catalase levels and complements could reflect association between inflammation and oxidative stress (Chen et al., 2012; Sellami et al., 2021b); however, the lower levels in younger high endurance athletes may reflect a protective mechanism against oxidative stress with high endurance exercise in younger, but not older, elite athletes.

### C2 and C3b/iC3b were negatively correlated with telomere length in younger elite athletes

Telomeres are repetitive nucleotide sequences located at the ends of chromosomes, which prevent loss of genetic information during replication. A shorter telomere length is a strong indicator of cellular ageing and potentially health decline (Shammas, 2011). Studies have shown that that young elite athletes have longer telomeres than their inactive peers (Tafere et al., 2020). Moderate chronic exercise was previously shown to be associated with longer telomeres and better health status and lifespan (Saygin et al., 2006; Wylleman and Reints, 2010). Endurance, but not resistance, training was also suggested to acutely increase telomerase activity and telomere length (Tafere et al., 2020), perhaps due to an acquiring an adaptive mechanism contributing to maintenance of telomeres (Saygin et al., 2006). Moderate exercise was too shown to be associated with changes in lengths of telomeres due to lowering of oxidative stress and inflammation (Sellami et al., 2021b). Furthermore, literature has suggested a strong correlation between VO_2_max and telomere length (Chen et al., 2012). In order to investigate whether younger elite endurance athletes have less cellular aging compared to their older counterparts, telomere lengths were determined in circulating blood cells. Our data have shown that lower levels of C2 and C3b/iC3b were associated with longer telomeres in younger elite athletes, which could reflect lower chronic inflammation and oxidative stress and elevated telomerase activity in younger elite athletes compared to older counterparts (Samjoo et al., 2013).

### Lower C3b/iC3b, C4b, C5, C5a, C1q, C3, C4, factor H and properdin in younger high endurance athletes

Lower levels of complements that belong to classical, alternative and lectin pathways were detected in younger high endurance elite athletes (Figure 5). This may suggest that lower inflammatory and oxidative stress status is associated with endurance training only in young athletes compared to their older counterparts or age-matched peers who belong to lower intensity sports (Samjoo et al., 2013). Our data agree with previous studies suggesting a significant decrease in C3 and C4 proteins in response to physical training in both athletes and non-athletes (Karacabey et al., 2005; Saygin et al., 2006). Indeed, aerobic exercise on a treadmill for 30 min was found to trigger a significant decrease in C3 and C4 proteins (Karacabey et al., 2005) and a similar post-game observation was reported in rugby players (Mashiko et al., 2004). Conversely, other studies have shown that short-term aerobic exercise activated C3 and C4 complement components and subsequently C3a and C4a (Smith et al., 1990). Furthermore, a post-effort increase in C3 and C4 complement components was observed in Marathon runners and their sedentary counterparts performing graded exercise on a mechanical treadmill (Nieman et al., 1989; Mochizuki et al., 1999). A similar trend was also observed in the case of C4 in older participants performing aerobic exercise (Kostrzewa-Nowak et al., 2020). Taken both opposing views into account, it may be concluded that endurance effort exerts alterations in the complement system that may depend on the intensity, duration and type of physical training.

Previous studies have also shown different effect of endurance effort on complement levels in athletes who belong to different age groups. For example, a decrease in the post-test C2 was only seen in younger participants (Kostrzewa-Nowak et al., 2020). It remains to be investigated whether age-related dysregulation of mitochondrial biogenesis, dynamic and autophagy could explain the compromised role of exercise on lowering complements levels in older athletes (Joseph et al., 2016; Sellami et al., 2021b). Comparing exercise-triggered mitochondrial alterations in relation to complement secretion in younger versus older athletes could be instrumental to understand the molecular pathways underlying age-dependent changes in the levels of circulating complements in elite endurance athletes.

## Study limitations

The relatively small number of participants and the diversity of their sport groups are main limitations of this study. The overrepresentation of females in the high endurance group represents another limitation of the study, although gender was corrected for in statistical analyses. Additionally, variation in timing of sample collection, transportation and storage could have also affected the results (Shivshankar et al., 2020), although standard operating procedures and shortest duration of sample delivery (within hours of collection) are usually implemented. Furthermore, the limited available information of athletes’ anthropometric, medical history, smoking habits, physiological and nutritional data during sampling as well as the resting time since their last exercise has hindered attempts to consider other important potential confounders such as body mass index and diet in our analysis. However, since samples were selected for doping tests, it suggests that the included participants were active elite athletes, hence unlikely suffering from any inflammatory or metabolic diseases, nor likely to be smokers or under chronic medication. Although various studies have investigated the effect of acute exercise on complement levels and despite all these limitations, our novel data has revealed significant and pronounced alterations in complements in the studied endurance and age groups. However, replication of data in other cohorts with controlled experimental design is warranted.

## Conclusion

Understanding the complement system in response to long-term low/moderate and high endurance exercise could provide insight into the mechanisms of exercise immunology and potentially grants a stronger molecular basis for the prevention of exercise-associated injuries and illnesses. The level of complements reported in our study suggest that young high endurance elite athletes exhibit lower levels of circulating complements, associated with lower inflammatory, oxidative stress and cellular aging. Future studies are warranted to investigate the health profile, performance and susceptibility to injuries in this group of elite athletes compared to their older peers or those who belong to lower intensity sports.

## Data Availability

The original contributions presented in the study are included in the article/Supplementary Material, further inquiries can be directed to the corresponding author.
